# Building Kinetic Models for Determining Vitamin C Content in Fresh Jujube and Predicting Its Shelf Life Based on Near-Infrared Spectroscopy

**DOI:** 10.3390/s131115673

**Published:** 2013-11-15

**Authors:** Yaohua Hu, Cong Liu, Qian Hao, Qiang Zhang, Yong He

**Affiliations:** 1 College of Mechanical and Electronic Engineering, Northwest A&F University, Yangling 712100, China; E-Mails: huyaohua@nwsuaf.edu.cn (Y.H.); liu_cong19880728@163.com (C.L.); haoqian@nwsuaf.edu.cn (Q.H.); 2 Department of Biosystems Engineering, University of Manitoba, Winnipeg, MB R3T 5V6, Canada; E-Mail: zhang@cc.umanitoba.ca; 3 College of Biosystems Engineering and Food Science, Zhejiang University, Hangzhou 310058, China

**Keywords:** fresh jujube, vitamin C content, kinetic model, shelf life, near-infrared spectroscopy

## Abstract

Fresh jujube (*Ziziphus jujube*) is rich in vitamin C, which is an important quality index and generally decreases with storage time. The aim of this study was to build kinetic models for determining the vitamin C content, thus predicting the quality characteristics and shelf life of fresh jujube. The quality changes of the jujube stored at room temperature (20 °C) were analyzed using near-infrared spectroscopy. The significant spectra were determined and a calibration model for vitamin C content was developed. The results showed that vitamin C content could be described by the zero-order kinetics model based on the regressions. In addition, the shelf life of the jujube at room temperature was calculated according to the regression model.

## 1. Introduction

Jujube (*Ziziphus jujube*) has been planted in China for a long time due to its excellent taste and high nutritional value, in particular the vitamin C content. However, the content of vitamin C in fresh jujube decreases sharply upon storage because of decay and oxidation [1], and the loss of vitamin C might be a critical factor for the shelf life of some products [2]. Therefore, it is important to develop rapid, reliable methods to detect the vitamin C in fresh jujube to achieve real-time monitoring in storage.

Much research has been reported on the chemical kinetic models of various products during storage, including seafoods, vegetables and juices [3–5]. Some researchers have developed first-order kinetic models for degradation of vitamin C in juice during storage [2]. However, there is little information available in the literature about the kinetics of vitamin C content in fresh jujube during storage.

Near-infrared (NIR) spectroscopy has recently attracted a great deal of attention as a non-destructive method for detecting the internal quality of fruits. A vast amount of research has been conducted using NIR spectroscopy to detect the quality of fruits, such as taste characterization in Valencia oranges [6]; soluble solids content (SSC) and firmness in apples [7]; acidity, soluble solids and firmness in Satsuma mandarin [8]; and vitamin C in Navel orange [9]. Van *et al.* [10] applied kinetic and near-infrared models to describe firmness and moisture loss with time change at constant external condition, respectively.

The objectives of this study were: (1) to optimize the NIR spectroscopy model of the vitamin C content of fresh jujube to predict the vitamin C content changes during storage at room temperature; (2) to build a kinetic model of vitamin C content according to NIR spectroscopy and storage time at room temperature; and (3) to predict the shelf life of fresh jujube at room temperature.

## 2. Material and Methods

### 2.1. Sample Preparation

Seventy-two intact “Lizao” jujube fruits weighing 25–30 g were hand-harvested from an orchard in Dali, Shaanxi Province, China in the year 2012. The 72 samples were labeled, randomly divided into 18 groups and put on the tray, then stored at room temperature (20 °C). The weight of each sample was measured and recorded before spectroscopic measurements. One group of samples was selected every day for the experiment. In other words, the experiment lasted for 18 days since the experiential decay period at room temperature is 15 days).

### 2.2. Spectral Measurements

Spectra were measured by using a Fourier transform near-infrared spectrometer (BRUKER MPA, Ettlingen, Germany) equipped with the OPUS 6.5 optical software. The spectrometer was allowed to warm up for about 30 min, and all spectra were obtained using a fiber optic probe with a wavenumber range of 12,000–4,000 cm^−1^ at 8 cm^−1^ intervals and a scan number of 64. A spectral white panel was used as reference to eliminate interference by the light source itself. The samples were divided to 18 groups. Every group had four samples. At the first day, the spectra of 72 samples were obtained, and the four samples of group one were adopted to measure the vitamin C content. At the second day, the spectra of the remaining 68 samples were obtained, and the four samples of group two were adopted to measure the vitamin C content. At the 18th day, only four samples were left. The spectra of the remaining four samples were obtained to measure the vitamin C content. Therefore, the samples of group one have one spectrum, the samples of group two have two spectra, the samples of group three have three spectra, *etc.* the samples of group eighteen have eighteen spectra. Moreover, three positions of each sample were measured for obtaining spectra. The average spectra of the three positions were adopted to decrease the error.

### 2.3. Determination of Vitamin C Content of Fresh Jujube

The vitamin C content of four jujube samples was measured every day right after the spectral measurement by using the 2,6-dichloroindophenol titration method [11]. The specific steps were as follows: 10 g of pulp of each sample were mashed with 10 mL extraction solvent (2% metaphosphoric acid and 2% oxalic acid), and then the mixture was put into a 100 mL volumetric flask after passing through a filter. One (1) g of activated carbon was added to prevent color interference. Ten (10) mL of filtrate was transferred to a 50 mL conical flask and titrated with 2,6-dichloroindophenol solution until it stayed pink for 15 s. The blank sample was used as reference. The vitamin C concentration was determined by the following equation:

(1) $$\text{VCC}=\frac{(V-V_{0})\cdot T\cdot A}{W}\times 100$$

where *VCC* = vitamin C concentration (mg/100 g); *V* = dye solutions volume of titrating liquid sample cost (mL); *V_0_* = dye solutions volume of titrating blank liquid cost (mL); *T* = 2,6-dichloroindophenol titer (mg/mL); *A* = dilution multiple; *W* = sample weight (g).

### 2.4. Spectral Data Analysis

The spectral data of each jujube measured at three locations were averaged. The pretreatment techniques of Savitzky-Golay smoothing (S-G smoothing, with the smooth points of left and right were 10), multiple scattering correction (MSC), first derivative (1-Der with the smooth points of left and right were 10) and second derivative (2-Der, with the smooth points of left and right were 10) were used to remove and reduce the noises and other measurement errors in the spectra data. In order to identify the optimal pretreat techniques, Savitzky-Golay smoothing (S-G smoothing), first derivative (1-Der), second derivative (2-Der) were compared with the raw spectra by using a statistical software package (Unscrambler 9.8, CAMO, CAMO Software AS, Oslo, Norway).

Multivariate linear regression (MLR) was chosen as the near-infrared modeling method. MLR can establish the relationship between the dependent and independent variables with simple calculations and clear physical meaning, and additionally it could be calculated by simply choosing the characteristic wavenumber absorption values corresponding to the composition. At the same time the predictive performance quality can directly reflect the prediction correlation of input variable (spectral data) to the chemical index. This study intends to use regression coefficient (RC) method as wavenumber selection method to choose spectra for ensuring the accuracy of the modeling and the quickness of operation.

All fresh jujubes were divided into two sample sets, 50 samples were randomly selected as the calibration (sample) set and 17 samples were randomly selected as the prediction set in the 67 samples (5 decayed samples were eliminated) in this experiment. The calibration sample set was used to establish a stable and reliable model, and the prediction set was used to examine the model performance.

### 2.5. Development of Model Based on Chemical Kinetics

Food quality-related changes are chemical, physical and microbial changes, which can be described by kinetics model chemical reactions. Changes in food quality in storage may be related to the kinetic characteristics, such as the reaction rate constant and activation energy. Most quality food changes follow zero-order or first-order reaction models [12], as described by the following equations:

(2) $$\text{Zero}-\text{order reaction}:C=C_{0}-Kt$$

(3) $$\text{First}-\text{order reaction}:C=C_{0}\times {\text{e}}^{-Kt}$$

where *C* = quality factor, *C_0_* = initial value of the quality factor, *t* = storage time, *K* = reaction rate constant.

Using the NIR model, the initial contents of vitamin C of fresh jujubes were calculated. The kinetic model could be developed based on the predicted initial contents, measured contents and storage time. This was achieved using the statistical program SPSS 18 (IBM, Armonk, NY, USA).

## 3. Results and Discussion

### 3.1. Pretreatment of Spectral Dada

The MLR technique was used to establish a model to correlate the vitamin C content to the NIR data. The pretreatment techniques such as S-G smoothing, MSC, 1-Der and 2-Der are used to remove the noise and other factors which are included in the spectra [13]. The S-G smoothing and MSC process spectra had a similar curve shapes as the raw spectrum, while the 1-Der and 2-Der processed spectra had different curve shapes from the raw spectrum.

The results showed that the spectra with MSC pretreatment technique better predicted the vitamin C content than did other spectra, and the model based on that technique did not show any under-fitting and over-fitting phenomena, and had a small degree of dispersion. Because of sample values changed from 3.250 mg/100 g to 398.287 mg/100 g in storage, the RMSEP was relatively large. This showed that the scattering from the uneven distribution of fresh jujube sample internal particles and particle size had an effect on the near-infrared spectra (Table 1). The MSC was selected as the pretreatment technique.

### 3.2. Determination of Spectral Regions

The absorption peaks were dissimilar due to the difference in functional groups. Six effective wavenumbers in the VNIR (12,000–4,000 cm^−1^) spectra, one in the SW-NIR (12,000–9,091 cm^−1^) spectra, and five in the LW-NIR (9,091–4,000 cm^−1^) spectra were used in the MLR analysis to develop the NIR models (Table 2). The correlation coefficient (R) of the VNIR and LW-NIR spectra models were higher than that in the SW-NIR one. The LW-NIR spectra model was selected for its higher predictability. Also the lower wavenumber is less demanding on the instrumentation, and thus lower cost. The effective wavenumbers were 8,330, 6,900, 5,666, 5,150 and 4,060 cm^−1^ in the LW-NIR range. The above ranges contain the combination frequencies of symmetric and antisymmetric stretching vibrations of water molecules, including the 10,300, 6,900 and 5,150 cm^−1^ regions, the relatively weak combination frequency absorption in the 4,710 cm^−1^ region, whereas the peaks at 5,666 and 4,060 cm^−1^ are normally attributed to CH and CONH_2_ functional groups, respectively [14].

The predictive model for the vitamin C content has the form of:

(4) $$\text{VCC}=1409.098+1586.574X_{1}+2145.536X_{2}-3501.009X_{3}-277.794X_{4}+30.116X_{5}$$

where *VCC* is the vitamin C content of fresh jujube, and *X_1_*–*X_5_* are the absorbance of the five effective wavenumbers in the LW-NIR range. The upper bound and lower bounds of the mean value for the 95% confidence interval were 173.683 and 217.234.

### 3.3. Change of Vitamin C Content of Fresh Jujube during Storage

**Figure 1.**
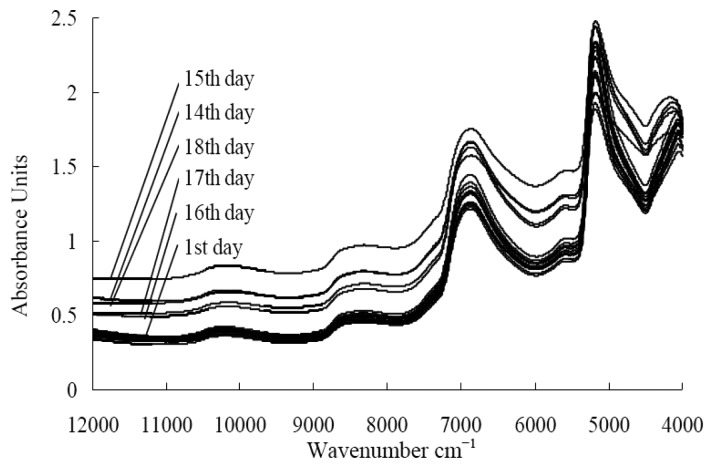
Spectra of fresh jujube in 18 days at room temperature.

The spectral curves of fresh jujube displayed obvious changes during storage. The absorbance increased with the storage time and it became a clutter during the last four days (Figure 1). The spectral curves for the 14th, 15th and 18th days were out of order due to the rotten jujubes.

**Figure 2.**
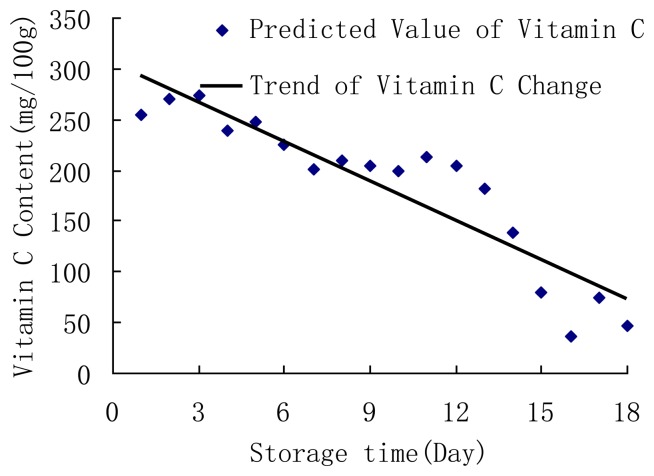
Predicted value of vitamin C of fresh jujubes after 18 days at room temperature.

The initial value of vitamin C of fresh jujube at room temperature could be calculated by using the NIR model and the spectra of each fresh jujube from the first day (Figure 2). Based on the measured vitamin C content and the initial value of vitamin C of fresh jujube that was predicted by NIR model, the kinetic models of vitamin C for jujube were determined as follows:

(5) $$\text{Zero order}:\text{VCC}=338.787+0.044{\text{VCC}}_{0}-20.677t$$

(6) $$\text{First order}:\text{VCC}=550.58{\text{e}}^{-0.164t}$$

where *VCC* is the vitamin content in mg/100 g after storing for *t* days, *VCC_0_* is the initial vitamin content in mg/100 g, and *t* is the storage time in days. The upper bound and lower bounds of the mean value for the 95% confidence interval were 150.648 and 201.676.

**Figure 3.**
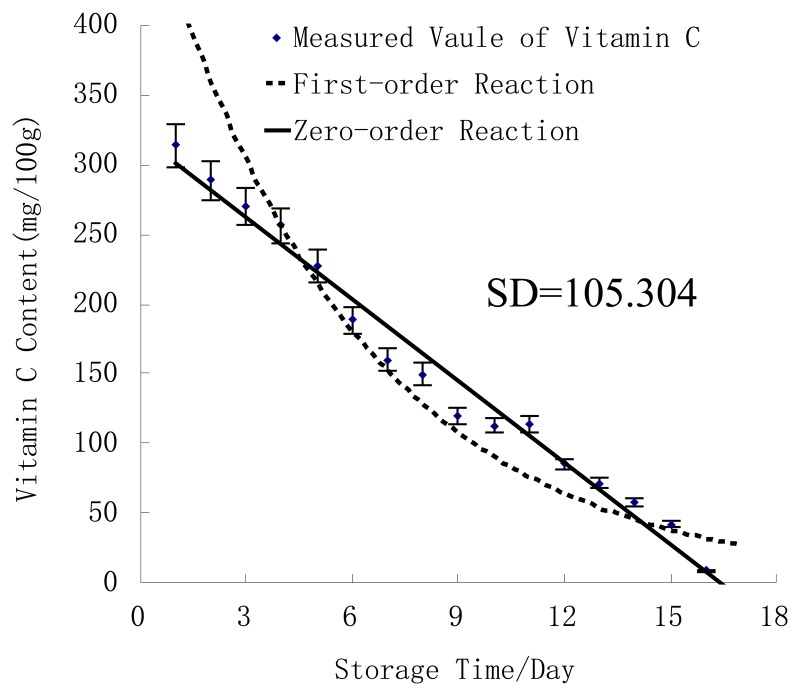
Kinetics models established by zero-order and first-order reactions.

Figure 3 shows the comparison of the two models. The zero-order reaction model had a higher correlation coefficient than the first-order reaction one (Table 3). It seemed reasonable to conclude that the change of vitamin C content of fresh jujube followed a zero-order kinetics reaction.

By substituting Equation (4) into Equation (5), the vitamin C content may be predicted directly from the NIR spectroscopy as follows:

(7) $$\text{VCC}=338.787+0.044\;(1409.098+1586.574X_{1}+2145.536X_{2}-3501.009X_{3}-277.794X_{4}+30.116X_{5})-20.677t$$

### 3.4. Determination of Safe Storage Time

The safe storage time could be predicted by the NIR with the kinetics model. Based on Equation (5), the equation of storage time is as follows:

(8) $$t=17.128-0.002{\text{VCC}}_{0}-0.046\text{VCC}$$

The maximum shelf life could be considered as 15 days when the vitamin C content decreased to about zero. Combing with NIR model (4), the equation could be described as:

(9) $$t=17.128-0.002\times (1409.098+1586.574X_{1}+2145.536X_{2}-3501.009X_{3}-277.794X_{4}+30.116X_{5})-0.046\times (1409.098+1586.574X_{1t}+2145.536X_{2t}-3501.009X_{3t}-277.794X_{4t}+30.116X_{5t})$$

where *X_1_*–*X_5_* are the absorbance of the effective wavenumber for fresh jujubes; and *X_1t_*–*X_5t_* are the absorbance of the effective wavenumber of spectra for jujubes storage for *t* days.

## 4. Conclusions

The vitamin C content of fresh jujube stored at room temperature could be predicted by NIR spectroscopy. The NIR model developed with the first derivative technique in the SW-NIR range better predicted the vitamin C content (correlation coefficient of 0.775) than did the S-G smoothing, the 1-Der and the 2-Der methods. The change in vitamin C content of fresh jujube stored at room temperature followed a zero-order kinetics reaction equation, and the correlation coefficient of the kinetic model was 0.977. Combining NIR with storage time the kinetics model could be used as a nondestructive method for determining of the vitamin C content of fresh jujube. The safe storage period could be predicted by applying the NIR kinetics model. The shelf life of fresh jujube when the vitamin C content decreased to about zero at room temperature was 15 days.

## Figures and Tables

**Table 1.** Result of different pretreatment technique applied in models.

**Pretreatment Technique**	**N**	**Rc**	**RMSEC**	**Rp**	**RMSEP**
**Raw Spectra**	6	0.876	93.517	0.741	98.117
**S-G Smoothing**	6	0.878	93.563	0.751	97.826
**MSC**	6	0.895	90.053	0.708	90.300
**1-Der**	12	0.942	100.369	0.915	97.653
**2-Der**	10	0.907	96.684	0.866	95.526

N: The number of characteristic wave number; R: Correlation coefficient; RMSEC: The root mean square error of calibration; RMSEP: The root mean square error of prediction.

**Table 2.** Result of different spectral region in models.

**Wavenumbers (cm**^−^**^1^)**	**N**	**RC**	**RMSEC**	**RP**	**RMSEP**
**12,000**–**4,000**	6	0.895	90.053	0.708	90.300
**12,000**–**9,091**	1	0.099	11.000	−0.627	1.028
**9,091**–**4,000**	5	0.775	85.735	0.620	76.169

**Table 3.** Result of kinetics models with vitamin C content of fresh jujubes at room temperature.

**Items**	**Zero-Order Reaction**	**First-Order Reaction**
**Correlation**	linear	exponent
**R**	0.977	0.782
**RMSEC**	98.954	130.049
**Kinetics Model**	*VCC* = 338.787 + 0.044*VCC_0_* − 20.677*t*	*VCC* = 550.58e^−^*^0.164t^*

## References

[1] Wang Y.Q., Li J.H., Zhao M., Feng J., Wang C.S. (2000). A study on changing law of vitamin C in fresh jujube fruit during storage. Act Agric. Boreali-Sin..

[2] Burdurlu H.S., Koca N., Karadeniz F. (2006). Degradation of vitamin C in citrus juice concentrates during storage. J. Food Eng..

[3] Yao L., Luo Y.K., Sun Y.Y., Shen H.X. (2011). Establishment of kinetic models based on electrical conductivity and freshness indictors for the forecasting of crucian carp (*Carassius carassius*) freshness. J. Food Eng..

[4] Giannakourou M.C., Taoukis P.S. (2003). Kinetic modelling of vitamin C loss in frozen green vegetablesunder variable storage conditions. Food Chem..

[5] Derossi A., Pilli T.D., Fiore A.G. (2010). Vitamin C kinetic degradation of strawberry juice stored under non-isothermal conditions. LWT Food Sci. Technol..

[6] Jamshidi B., Minaei S., Mohajeraui E., Ghassemian H. (2012). Reflectance Vis/NIR spectroscopy for nondestructive taste characterization of Valencia oranges. Comput. Electron. Agric..

[7] Bobelyn E., Serban A.S., Nicu M., Lammertyn J., Nicolai B.M., Saeys W. (2010). Postharvest quality of apple predicted by NIR-spectroscopy: Study of the effect of biological variability on spectra and model performance. Postharvest Biol. Technol..

[8] Gomez A.H., He Y., Pereira A.G. (2006). Non-destructive measurement of acidity, soluble solids and firmness of Satsuma mandarin using Vis/NIR-spectroscopy techniques. J. Food Eng..

[9] Xia J.F., Li X.Y., Li P.W., Ma Q., Ding X.X. (2007). Application of wavelet transform in the prediction of navel orange vitamin C content by near-infrared spectroscopy. Agric. Sci. China.

[10] Dijk C.V., Boeriu C., Peter F., Smits T.S., Tijskens L.M.M. (2006). The firmness of stored tomatoes (cv. Tradiro). 1. Kinetic and near infrared models to describe firmness and moisture loss. J. Food Eng..

[11] National Bureau of Standards of China (1986). GB/T 6195—86 Determination of vitamin C in vegetables and fruits (2,6-dichloro-indophenol titration method)..

[12] Martinus A.J.S., Bookel V. (2008). Kinetic modeling of food quality: A critical review. Food Sci. Food Saf..

[13] Nicolai B.M., Beullens K., Bobelyn E., Peirs A., Saeys W., Theron K.I., Lammertyn J. (2007). Nondestructive measurement of fruit and vegetable quality by means of NIR spectroscopy: A review. Postharvest Biol. Technol..

[14] Workman J., Weyer L. (2009). Practical Guide to Interpretive Near-Infrared Spectroscopy.

